# HMGCS2 is a key ketogenic enzyme potentially involved in type 1 diabetes with high cardiovascular risk

**DOI:** 10.1038/s41598-017-04469-z

**Published:** 2017-07-04

**Authors:** Sanket Kumar Shukla, Weijing Liu, Kunal Sikder, Sankar Addya, Amrita Sarkar, Yidong Wei, Khadija Rafiq

**Affiliations:** 1 Department of Medicine, Center of Translational Medicine, Thomas Jefferson University, Philadelphia, PA-19107 USA; 2 Internal Medicine-Cardiovascular Department, Shanghai Tenth People’s Hospital, Middle of Yanchang Road, Zhabei district Shanghai, China; 3 Kimmel Cancer Centre, Thomas Jefferson University, Philadelphia, PA-19107 USA; 4 Department of Cardiology, Shanghai Tenth People’s Hospital of Tongji University, 301 Yanchang Road, Shanghai, China

## Abstract

Diabetes increases the risk of Cardio-vascular disease (CVD). CVD is more prevalent in type 2 diabetes (T2D) than type 1 diabetes (T1D), but the mortality risk is higher in T1D than in T2D. The pathophysiology of CVD in T1D is poorly defined. To learn more about biological pathways that are potentially involved in T1D with cardiac dysfunction, we sought to identify differentially expressed genes in the T1D heart. Our study used T1D mice with severe hyperglycemia along with significant deficits in echocardiographic measurements. Microarray analysis of heart tissue RNA revealed that the T1D mice differentially expressed 10 genes compared to control. Using Ingenuity Pathway Analysis (IPA), we showed that these genes were significantly involved in ketogenesis, cardiovascular disease, apoptosis and other toxicology functions. Of these 10 genes, the 3-Hydroxy-3-Methylglutaryl-CoA Synthase 2 (*HMGCS2*) was the highest upregulated gene in T1D heart. IPA analysis showed that *HMGCS2* was center to many biological networks and pathways. Our data also suggested that apart from heart, the expression of HMGCS2 was also different in kidney and spleen between control and STZ treated mice. In conclusion, The *HMGCS2* molecule may potentially be involved in T1D induced cardiac dysfunction.

## Introduction

Cardiovascular disease (CVD) is one of the most common disease in patients with diabetes. Although the CVD is more prevalent in type 2 diabetes (T2D), but the mortality risk is higher in TID than in T2D^1, 2^. Most published work on CVD has been focused on the T2D and assumed that pathogenesis of CVD is similar to that in T1D. Despite hyperglycemia representing a common diagnostic phenotype between T1D and T2D, the two diseases are far more different^3^. Therefore, mechanism of CVD can also be different in both type of diabetes. Very little information is currently available regarding outcomes of T1D patients following CVD.

Hyperglycemia and metabolic disorder cause oxidative stress, which in turn causes overproduction of reactive oxygen species (ROS) and impaired antioxidant function of tissue. After long term exposure to ROS, tissue develops chronic inflammation and fibrosis which leads to cardiac dysfunction^4^. Numerous experimental studies found that cardiac dysfunction is associated with altered cardiac substrate and energy metabolism^5, 6^. In recent years’ investigators have reported that ketone bodies induce oxidative stress and upregulate several signaling pathways involved in diabetic complications^7, 8^. Metabolic and bioenergetics pathway indicate that ketone body metabolism could play an important role in the myopathic heart^9^. However, how ketone bodies affect the diabetic heart specifically in T1D is still not known. Moreover, the key molecules involved in this pathway still need to be identified.

Thus, in the current study, we performed genome-wide analyses in heart tissue of T1D mice model with cardiac dysfunction by means of microarray technique to identify novel molecules and pathways. By using Ingenuity Pathway Analysis, we have presented a detail bioinformatics analysis of a set of 10 genes selected from microarray data to evaluate the pathways and networks involved during the process of T1D associated CVD. Further, we identified hydroxymethylglutaryl (HMG)-CoA synthase 2 (HMGCS2) as the most upregulated gene and a key ketogenic enzyme for all biochemical pathways and networks in T1D mice with cardiac dysfunction. We have also presented a detailed expression of signaling pathways that could be regulated by HMGCS2 during the development of cardiac dysfunction in T1D.

## Material and Method

### Animals

C57BL/6 male mice, 8 to 10 weeks of age, were purchased from the Jackson Laboratory (Bar Harbor, Maine) and housed at Thomas Jefferson University at 22 °C with a 12-h light/dark cycle with free access to standard rodent chow and tap water. All animal protocols have been approved by the Institutional Animal Care Committee of Thomas Jefferson University, and experiments conformed to the *Guide for the Care and Use of Laboratory Animals* published by the U.S. National Institutes of Health and approved by the American Physiological Society. All the methods were carried out in accordance with the relevant guidelines and regulations.

T1D was introduced to the mice by injecting STZ [Sigma-Aldich, St. Louis, MO], dissolved in 0.1 M sodium citrate (pH 4.5) at 50 mg/kg body weight, i.p. for 5 consecutive days. While age-matched control mice received sodium citrate buffer injection in the same manner. Five days after the last injection of STZ, mice with hyperglycemia (blood glucose levels ≥250 mg/dl) were defined as diabetic as described previously^10^. Mice were sacrificed for experimental measurements at 8 weeks after STZ injection.

### Hemodynamic and echocardiography measurements

Echocardiographic measurements were done before STZ injection as well as at 8 weeks after STZ injection to determine the baseline heart function and ventricular dimensions in the experimental groups. Briefly, following light sedation with 1% isoflurane, the mice were placed on a heated platform in the left lateral decubitus position for imaging. A Visualsonic Ultrasound System (Vevo770, Toronto, Canada) containing a 40 Mhz variable frequency probe was used to capture the echocardiogram. All hair was removed from the chest using a chemical hair remover and the aquasonic clear ultrasound gel (Parker Laboratories, Fairfield, NJ) without bubbles was applied to the thorax surface to optimize the visibility of the cardiac chambers. Standard long and short axis M-Mode views were recorded when the mouse possessed a target heart rate between 450 and 550 beats per minute. Percent fractional shortening was calculated using:

$$ \% {\rm{FS}}=[({\rm{LVEDD}}-{\rm{LVESD}})/\mathrm{LVEDD}]\times 100.$$

LVEF was calculated using the cubed method:

$${\rm{LVEF}}=[{({\rm{LVEDD}})}^{3}-{(\mathrm{LVESD})}^{3}]/{(\mathrm{LVEDD})}^{3}.$$

### Microarray analysis

Total LV heart RNA was isolated from STZ-treated WT and age-matched control mice. RNA isolation was done using RNeasy mini kit (Quigen, Mansfield, MA), according to manufacturer instructions. The RNA quality measurement was performed as previously described^11^. Fragmented biotin labelled cDNA (from 100ng of RNA) was synthesized using the GeneChip WT PLUS reagent kit (Affymetrix, Santa Clara, CA). The protocols for microarray experiments were previously described^12^. In brief, Affymetrix gene chips, Mouse Gene 2.0 ST Array were hybridized with 4.5 µg fragmented and biotin-labeled cDNA in 200 µl of hybridization cocktail. Target denaturation was performed at 99 °C for 5 min and then 45 °C for 5 min, followed by hybridization for 16 h at 45﻿°﻿﻿C. Then the arrays were washed and stained using Genechip Fluidic Station 450, using Affymetrix GeneChip hybridization wash and stain kit. Chips were scanned on an Affymetrix Gene Chip Scanner 3000 7G, using Command Console Software.

### Quantitative real-time (qRT-PCR)

cDNA was generated from 1–5 µg of total RNA using High Capacity RNA-to-cDNA kit (Life Technology Grand Island, NY) according to the manufacturer’s instruction. qRT-PCR assays were performed using PerfeCT Super Green Super Mix (Quanta Biosciences, Beverly, MA) by using specific primers (Invitrogen, Carlsbad, CA) Differences in expression were determined by the relative quantification method; the cycle threshold (C_T_) values of the target genes were first normalized to the C_T_ values of endogenous control GAPDH.

### Ingenuity Pathway Analysis (IPA)

The list of differentially expressed genes was loaded into Ingenuity Pathway Analysis (IPA) 8.0 software (http://www.ingenuity.com) to perform biological network and functional analyses as reported previously^13^.

### Antibodies

The following antibodies were used for western blot. HMGCS2 antibody was purchased from Abcam (Cambridge, MA). pERK(Thr202/Tyr204), pAkt(S473),pP38(pT180/pY182), pJNK(p183/p185) and NFkB(p65,D14E12) antibodies were purchased from Cell Signaling Technology, (Danvers, MA). ERK, AKT and P38 antibodies were purchased from Santa Cruz Biotechnology (Dallas, Texas).

#### Ketone bodies measurement

Whole blood was isolated by cardiac puncture in mice model and serum were used to measure ketone bodies. The β-hydroxy butyrate (ketone body) level were measured by β-hydroxy butyrate calorimetric assay kit (Biovision Incorporated, Milpitas CA) as per manufacturer instruction.

### Statistical analysis of microarray data

Data analyses were performed by using GeneSpring software version 14.5 (Agilent Technologies, Inc., Santa Clara, CA). A paired t-test compared the “before” and “after” treatment samples. A statistical threshold of *p* < 0.05 with fold-change (FC) ≥ 2 was considered as significant for identification of differentially expressed genes. The probe set signals were calculated with the Iterative Plier 16 summarization algorithm; baseline to median of all samples was used as baseline option.

## Results

### Characterization of cardiac dysfunction in T1D mice model

**Figure 1:**
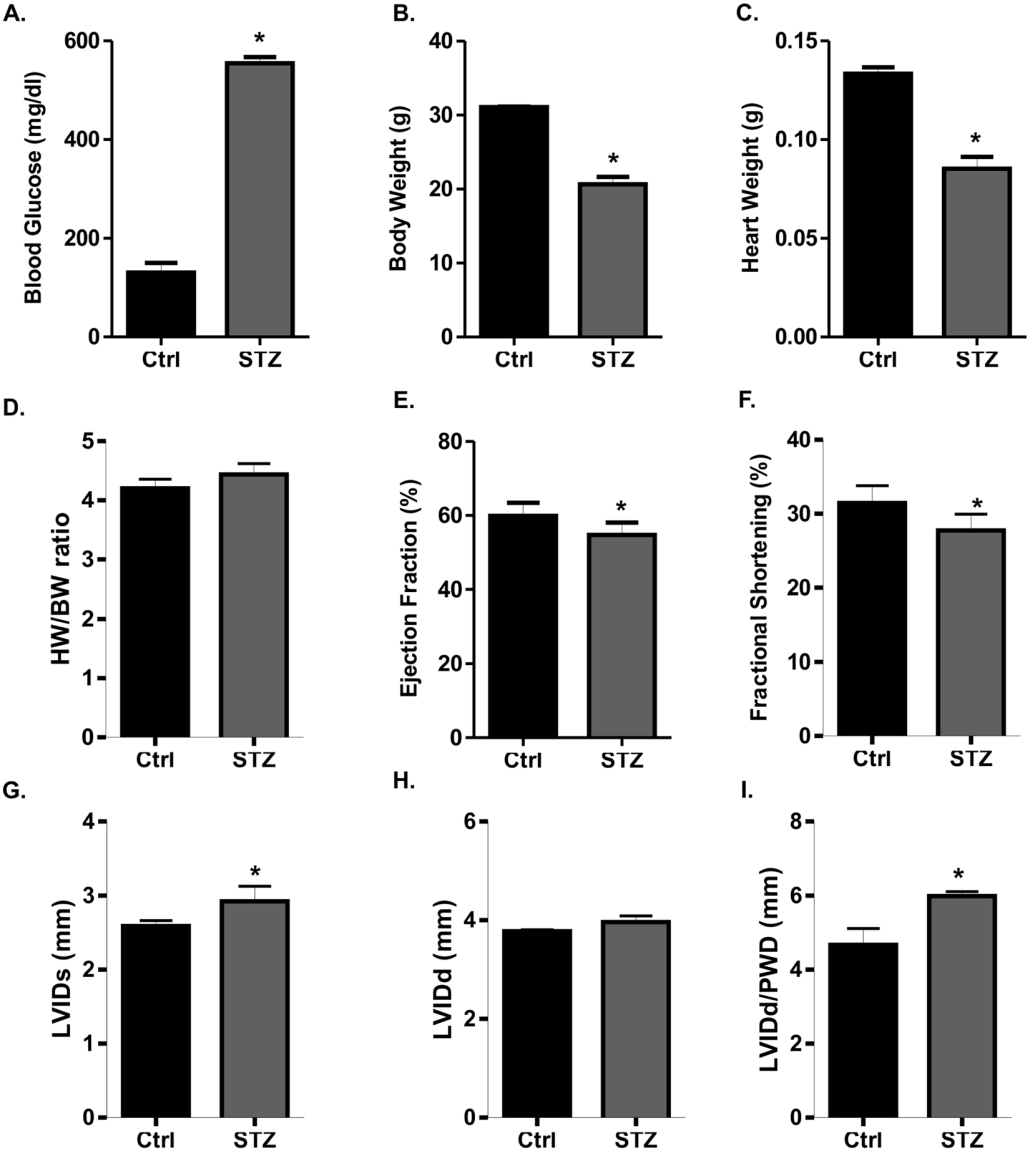
The morphological and echocardiography measurement in STZ treated mice. (**A**) Blood Glucose; (**B**) Body weight (BW); (**C**) Heart weight (HW); (**D**) HW/BW ratio in control and STZ treated mice. Echocardiographic analysis of (**E**) Ejection Fraction (EF); (**F**) fractional shortening (FS); (**G**) left ventricle internal diameter, systole (LVIDs); (**H**) left ventricle internal diameter, diastole (LVIDd); (**I**) LVIDd/PWD ratio in control and STZ treated mice (*P < 0.05).

T1D was confirmed in mice at 7 days after STZ injection based on a blood glucose level > 250 mg/dl. STZ-induced diabetic mice showed severe hyperglycemia that correlated with a decrease in body weight and heart weight, resulting in a slight increase of heart to body weight (HW/BW) ratio as compared to the non-diabetic control mice (Fig. 1A–D). Given these morphological findings, we next evaluated the cardiac function of STZ treated mice by echocardiography. Left ventricular ejection fraction (LV EF) and LV systolic function (Fractional Shortening; FS) were significantly reduced in the diabetic hearts (Fig. 1E,F). STZ treated diabetic mice had significantly increased left ventricular internal dimension in systole (LVIDs) (Fig. 1H). Although the left ventricular internal dimension in diastole (LVIDd) was higher in STZ treated mice but not significantly different from control (Fig. 1G). However, there was significant increase in LVIDd/PWD ratio in STZ treated mice compared to control (Fig. 1I).

### Gene expression profiling in heart tissues of STZ-induced T1D mice

**Figure 2:**
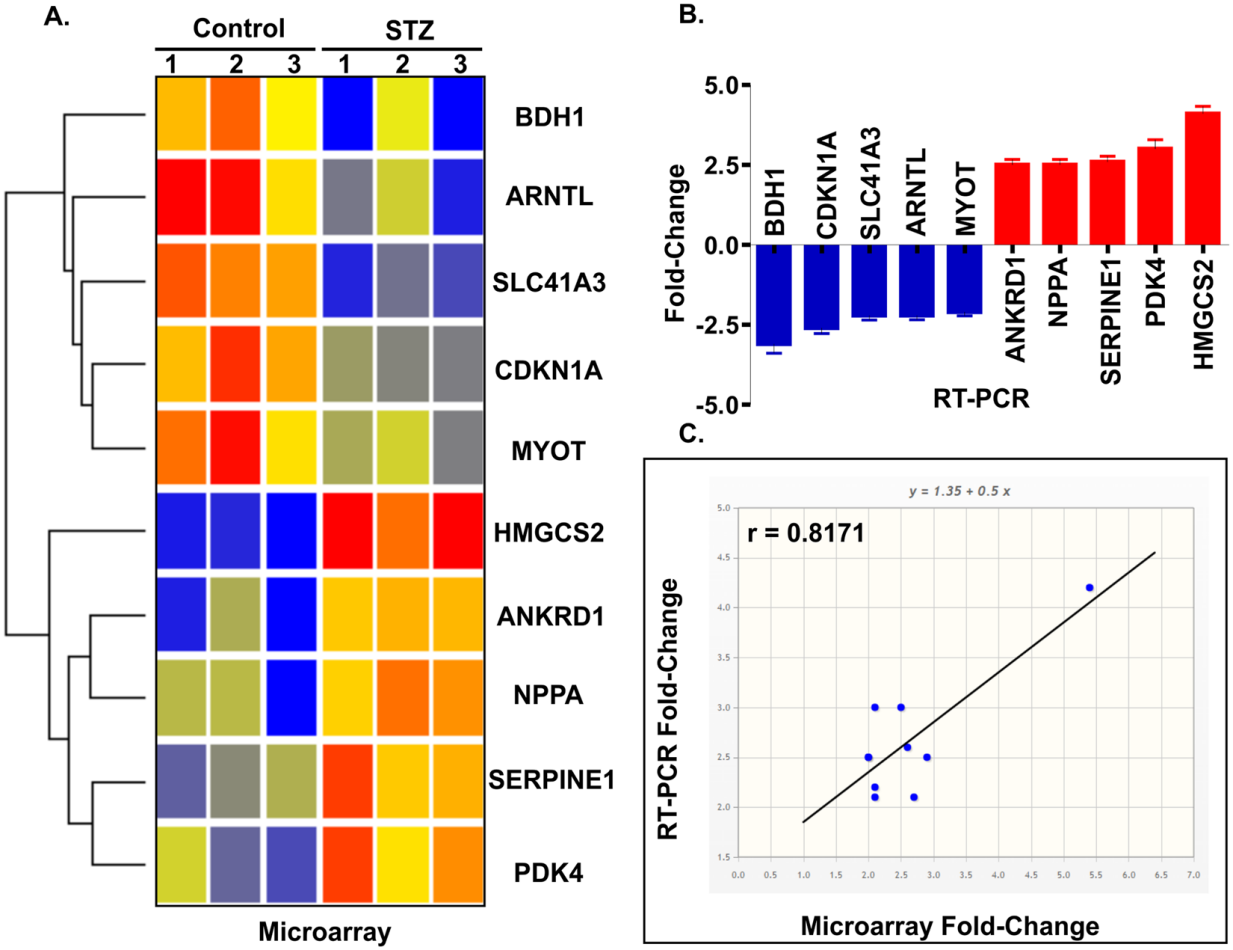
Co-differentially-expressed genes in STZ treated T1D mice with cardiac dysfunction. (**A**) Heat map represents data of all 10 differentially expressed genes in STZ-treated diabetic mice compared with control. Genes were identified by performing t tests on the large original dataset and comparing each diabetic group with the relative control group. Features with significantly different expression (P < 0.05) were included. The heat map is organized with individual samples arranged along the Y-axis, and the relative ratios of expression are indicated by color. Color intensity is scaled as highest expression corresponding to bright red and the lowest expression corresponding to bright blue. Genes that are up- or down-regulated ≥ 2-fold are displayed in red (up-regulation) and blue (down-regulation) respectively. (**B**) qRT-PCR expression bar diagram of all 10 differentially expressed genes. (**C**) Correlation in gene expression between microarray (x-axis) and qRT-PCR (y-axis) changes in gene expression with r = 0.8171; *P < 0.05.

Gene expression profiling was performed using Affymetrix gene chips, Mouse gene 1.0 ST array. By using a 2-fold cut off for changes in expression and a P value < 0.05, the chip array revealed that the total of 10 genes were differentially expressed in STZ induced T1D mice compared to control (Fig. 2A). These genes were either up- or down- regulated based on heat map after cluster algorithms (Fig. 2A).

We surveyed the literature to identify the functional significance of these 10 genes. Details on the function of these genes including p-values appear in Table 1. In summary, among the top 10 differentially regulated genes, *HMGCS2* was most upregulated (5.40 fold). *HMGCS2* is a mitochondrial enzyme that catalyzes the first reaction of ketogenesis, a metabolic pathway that provides lipid-derived energy during the fasting^14^. Other genes such as *ANKRD1* (ankyrin repeat domain 1), *PDK4* (Pyruvate dehydrogenase kinase, isoenzyme4), *NPPA* (Natriuretic peptide A) and *SERPINE-1* (Serpine peptidase inhibitor) were also found to be upregulated. We also found that some genes including *ARNTL* (aryl hydrocarbon receptor nuclear translocator like), *SLC41 A3* (solute carrier family 41 member 3), *BDH1* (3-hydroxybutyrate dehydrogenase, type 1), *CDKN1A* (cyclin-dependent kinase inhibitor 1A) and *MYOT* (Myotilin) were significantly downregulated in STZ induced TID.

**Table 1:** Function of top 10 genes up- or down-regulated in T1D associated with CVD.

Gene	Gene Product	Fold Change	P value	Function
**Up-regulated**				
HMGCS2	3-hydroxy-3-methylglutaryl-CoenzymeA synthase2	5.40	0.003	Ketogenesis
ANKRD1	Ankyrin repeat domain 1	2.12	0.007	Regulate myofibrillar stretch-sensor system
PDK4	Pyruvate dehydrogenase kinase, isoenzyme4	2.10	0.02	Inhibits conversion of pyruvate to acetyl CoA
NPPA	Natriuretic peptide A	2.05	0.02	Electrolyte homeostasis
SERPINE1	Serpin peptidase inhibitor	2.03	0.01	Serine proteinase inhibitor (serpin) superfamily
**Down-regulated**				
ARNTL	Aryl hydrocarbon receptor nuclear translocator like	2.89	0.03	Circadian clock
SLC41A3	Solute Carrier family 41 member 3	2.58	0.0007	Transmembrane Transport
BDH1	3-hydroxybutyrate dehydrogenase, type 1	2.53	0.04	Mitochondrial membrane enzyme
CDKN1A	Cyclin-dependent kinase inhibitor 1A (p21, Cip1)	2.12	0.01	Regulator of cell cycle progression at G1
MYOT	Myotilin	2.10	0.03	Stability of thin filaments during muscle contraction

Next, we confirmed our gene array data and observations by qRT-PCR analysis. We used the same samples in each time point as for the microarray analysis. To check the specificity of our microarray data, we performed qRT-PCR for all 10 differentially expressed genes. All of the reactions were performed in triplicate from total RNA samples from each specimen at each group. We found qRT-PCR expression profiles were highly similar to microarray data (Fig. 2B). Figure 2C illustrates the correlation between qRT-PCR and microarray data, with a resulting correlation coefficient of r = 0.8171(*P* < 0.05 at 95% confidence level).

### Bioinformatics analysis of the transcriptomic data

To gain insight into how T1D may affect the cellular functions and classes of molecules in cardiac dysfunction, we used the Ingenuity Pathway Analysis (IPA) software to perform a comprehensive analysis of the transcriptome variations.

#### Pathway analysis of differentially expressed genes in STZ induced T1D heart

**Figure 3:**
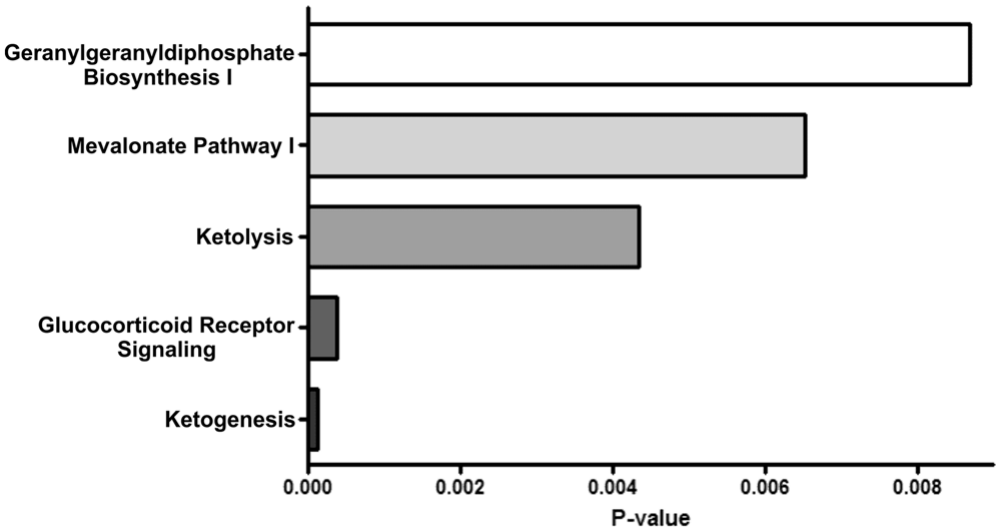
IPA canonical pathway analysis of differentially expressed genes in STZ-treated T1D mice with cardiac dysfunction. The bar diagram represents the top 5 over-represented canonical pathways in T1D associated with CVD. Category names are presented on the y-axis. The x-axis indicates the p value of the over-representation analysis. The inclusion of pathways is based on the presence of any 10 differentially regulated genes in DCM.

To further explore the differences between expressions of genes in T1D associated CVD, we compared the genes that were differentially expressed in the hearts of mice with STZ-induced T1D. The differentially expressed genes were more likely to belong to ketogenesis, glucocorticoid receptor, ketolysis and mevalonate pathways (Fig. 3).

#### *Functional annotation of genes differentially regulated in STZ-induced T1D heart*

**Figure 4:**
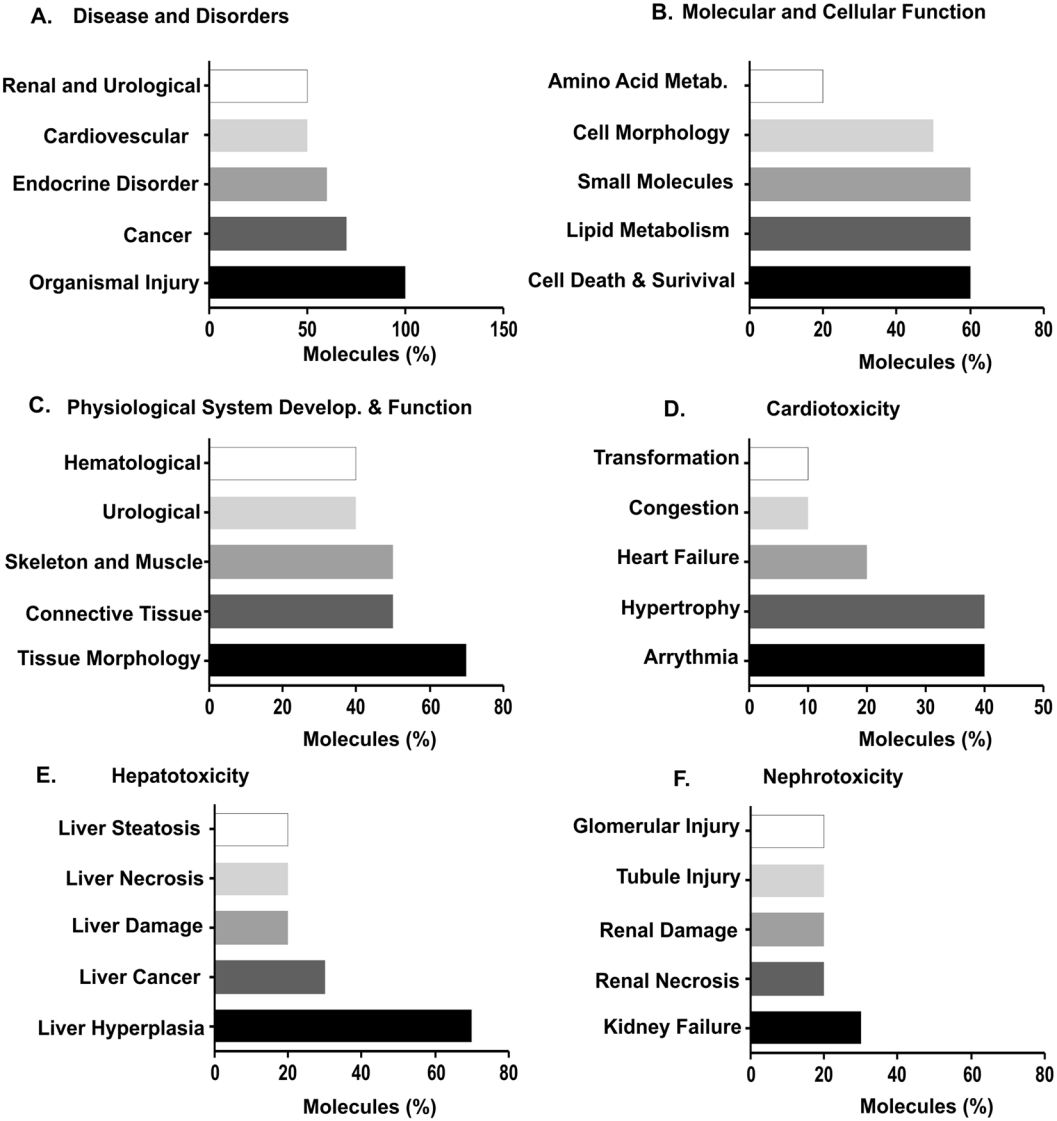
Top functions of over expressed genes in STZ treated T1D mice with cardiac dysfunction. (**A**) Top disease and disorders, (**B**) Top molecular and cellular function, (**C**) Top physiological system and development and function, (**D–F**) Organ-specific top toxicology function. The X axis represents the percentage of molecules among 10 differentially expressed genes involved in different functions.

The IPA comprised 2 structured networks: (1) disease and bio functions and (2) toxicity functions. The data was calculated based on the percentage of molecules which were significantly associated with all the functions. Through IPA analysis, we found that the differentially expressed mRNAs were principally enriched for various disease functions such as organismal injury, cancer, endocrine, cardiovascular disease as well as renal and urological disorders (Fig. 4A). The dysregulated genes were also associated with molecular and cellular functions such as cell death and survival, lipid and amino acid metabolism and cell morphology (Fig. 4B). The top physiological and development functions were tissue morphology, connective tissue, skeleton, urological and hematological development and function (Fig. 4C). We also analyzed the toxicology functions all differentially expressed genes and found these genes were involved in major organ specific toxicology functions such as cardiotoxicity, hepatotoxicity and nephrotoxicity functions (Fig. 4D–F).

#### Molecular Networks map generated by the Ingenuity Pathway Analysis tool

To examine the interaction of the differentially regulated genes with other gene products, we formed molecular networks in IPA using functional relationships. The molecular networks between gene products, based on known connections in peer-reviewed literature, revealed the molecules involved in different pathways, binding, activation, inhibition and biological process during T1D associated CVD.

**Figure 5:**
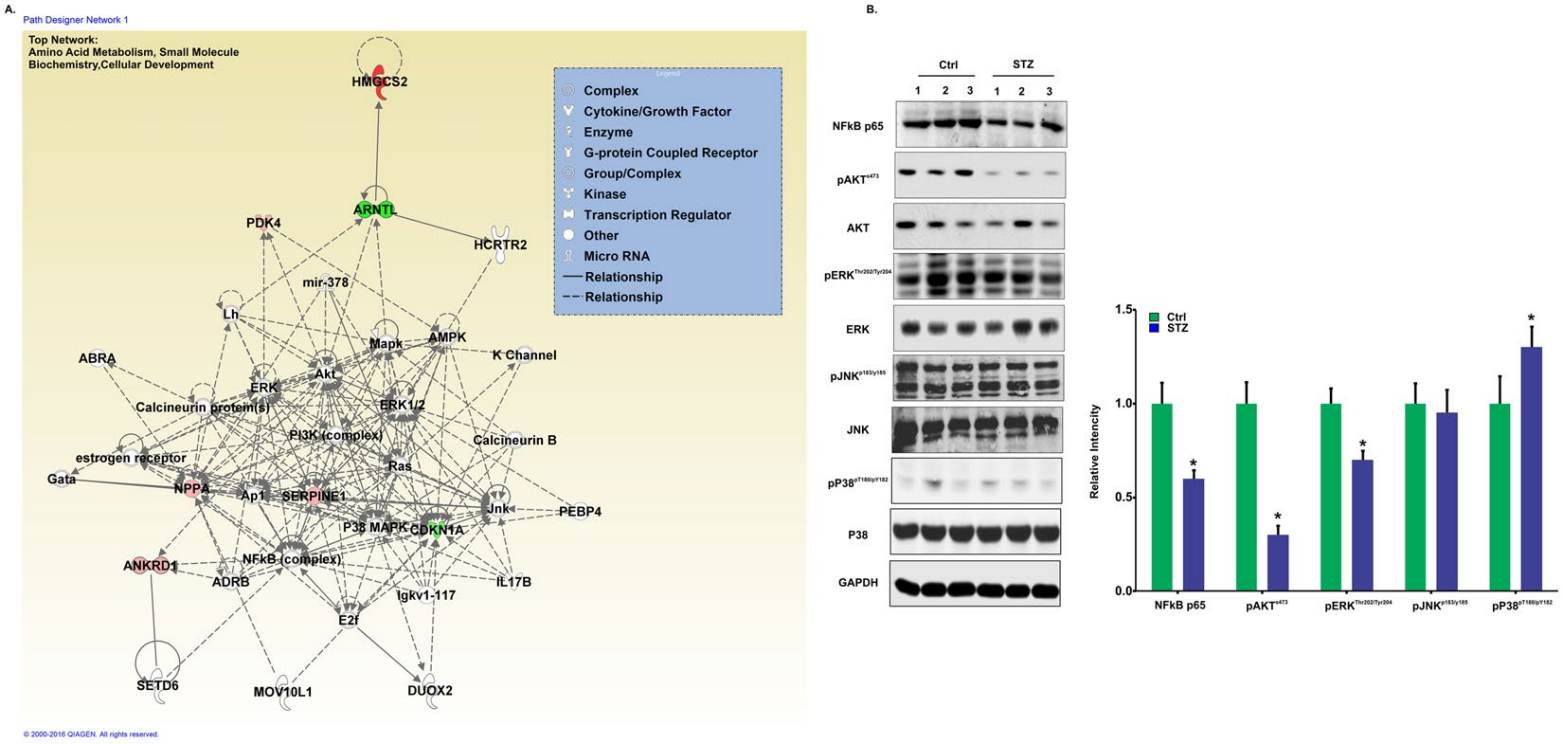
Functional gene networks identified using IPA for STZ-induced T1D with cardiac dysfunction. (**A**) Representative diagram of top network identified named as Amino acid Metabolism, Small Molecule Biochemistry, Cellular Development (IPA score 17). Nodes represent genes, whereas shapes represent a functional class of gene product. Intensity of the node color indicates the degree of overexpression (red) and the degree of down-regulation (green). Genes in uncolored nodes were not identified as differentially expressed and were integrated into computationally generated networks based on information in the IPA database. The network diagram was generated by Ingenuity® Pathway Analysis software (QIAGEN, Bioinformatics, Redwood City, CA), www.qiagenbioinformatics.com. (**B**) Differential expression profile of MAPKs and NFkB in heart tissue of the control and STZ treated mice.

All of the 10 differentially expressed genes were used in the IPA network analysis. Two main networks were identified. The top network with an IPA scores of 17 and includes genes and pathways involved in Amino Acid Metabolism, Small Molecule Biochemistry and Cellular Development (Fig. 5A). This network clearly showed that most of the differentially expressed genes are involved and regulated by different signaling pathways such and PI3K/AKT, MAPK/ERK and NFkB. Other top networks, with an IPA score of 7, include Cellular Development, Hematological System Development and Function, Hematopoiesis (Fig. S1).

We also performed upstream regulator analysis by IPA. This tool predicts which upstream regulators are activated based on our microarray expression data. Our data predict increased activation for upstream regulators AKT, DYRK1A, ZFYVE16, INSR and mir-30 (P < 0.05) contributed to T1D with cardiac dysfunction.

To explore the underlying signaling mechanisms for the above process we conducted a throughput analysis of several pathways that were present in IPA network. Figure 5B shows that after diabetes induction, AKT and ERK phosphorylation were decreased as compare to control. However, P38 phosphorylation was increased. These effects are critical for regulation of cadiac growth and survival. It is noteworthy, that the increase or decrease in AKT, ERK or P38 phosphorylation was not associated with an increase or decrease at the expression levels of these molecules at this stage of diabetes. We also found the down regulation of NFkB pathway in STZ treated diabetic mice (Figure 5B).

### Differential expression of HMGCS2 gene in various organs of T1D mice

**Figure 6:**
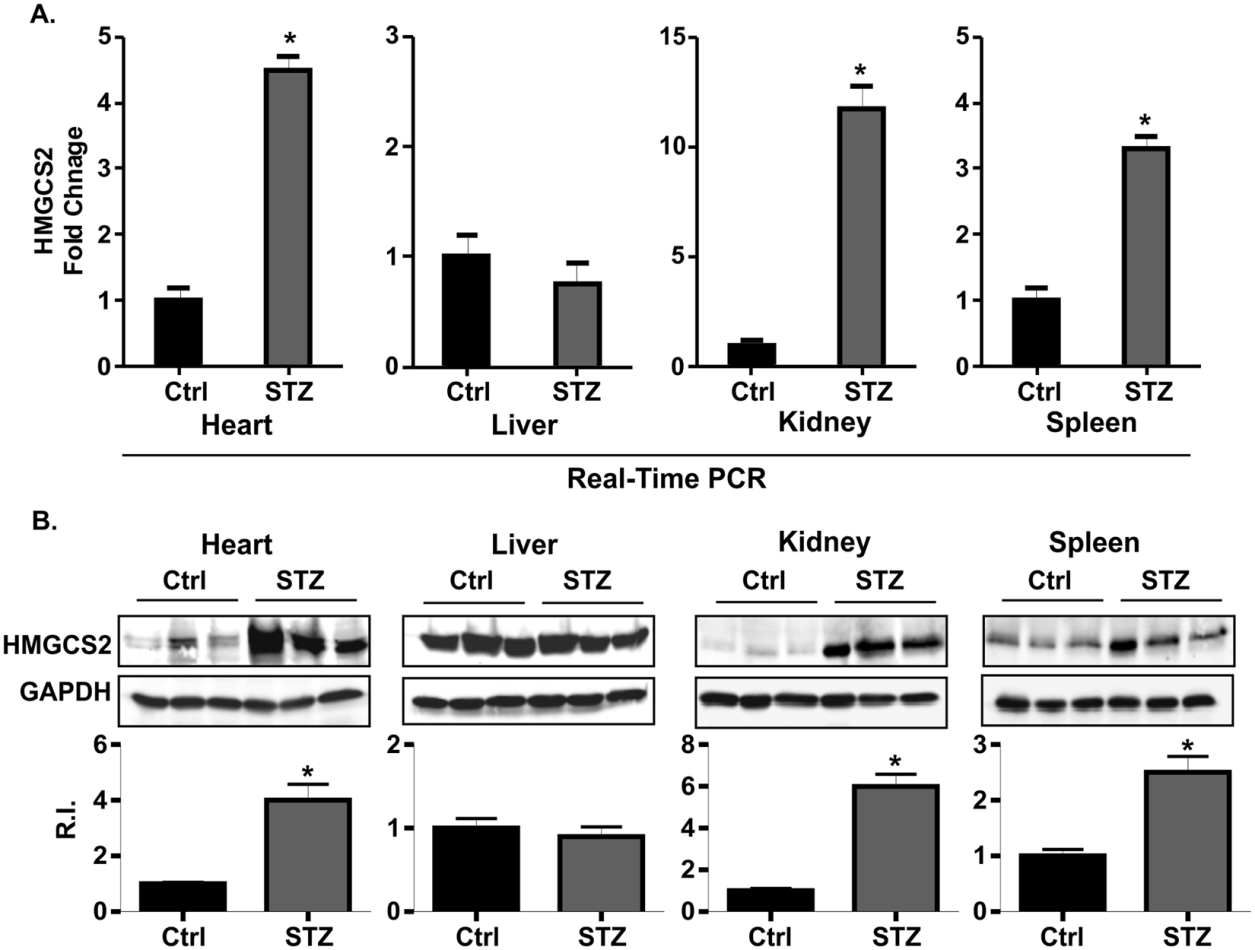
Expression profile of HMGCS2 in different tissues of STZ treated T1D mice with cardiac dysfunction. (**A**) The expression profile of HMGCS2 at mRNA level by real-Time PCR in heart, liver, kidney and spleen. (**B**) The expression profile of HMGCS2 at protein level by western blot in heart, liver, kidney and spleen.

In the current study, the most up-regulated gene HMGCS2 was 5.2-fold increased at the mRNA level. This increase was surprising since the HMGCS2 enzyme is typically present in the heart at early stages of perinatal development^15^. This molecule is generally involved in ketogenesis in the liver when high fatty acids are available^16^. Therefore, our next experiments were designed to determine whether HMGCS2 expression was increased by diabetes only in the heart or other vital organs such as the liver, kidney and spleen. The expression of HMGCS2 was checked both at mRNA and protein level by qRT-PCR and western blot respectively. The expression of HMGCS2 (both mRNA and protein levels) was significantly higher in heart, kidney and spleen tissue of T1D mice compared to control. A higher basal expression of HMGCS2 was detected in liver compared to other organs but no significant difference was observed in STZ treated mice compared to control (Fig. 6). Our next experiment was designed to determine whether HMGCS2 overexpression might correlated with ketone body synthesis. We measured β-hydroxy butyrate level in serum sample of mice model. An elevated level of β-hydroxy butyrate level was reported in T1D compared to control (Fig. S2). This result supports that HMGCS2 is a key molecule in T1D which is involved in ketone body synthesis.

## Discussion

The mechanisms responsible for T1D associated cardiac dysfunction are complex and poorly understood^17^. A previous concept has linked oxidative stress, inflammation and apoptosis to the maladaptive cardiac structural and functional consequences of diabetes associated cardiac dysfunction^18, 19^. Chronic hyperglycemia, induced by STZ, may prompt a cascade of complex pathophysiological events characterized by biochemical and functional myocardial abnormalities in mice, eventually leading to heart failure^20^. To define molecular mechanism, it is important to identify novel molecules and pathways for novel therapeutic approaches during the process of cardiac dysfunction in T1D.

Our most remarkable finding from our microarray analyses was that we identified differentially expressed genes associated with cardiac dysfunction in T1D. Among all 10 differentially expressed genes, HMGCS2 was the most upregulated gene. This enzyme is generally known to be involved in ketone synthesis pathways^21^. Although few studies have reported the expression of HMGCS2 in the heart tissue^22, 23^ none have investigated its role inT1D during the development of cardiac dysfunction. More recently Cook *et al*.^24^ reported that expression of HMGCS2 was higher in T1D rat^24^. However, this study did not adequately explore the cardiac dysfunction and pathway analysis for HMGCS2 during the development of T1D. To the best of our knowledge our study is the first to report that upregulation of HMGCS2 is associated with STZ induced T1D mice that lead to cardiac dysfunction. Moreover, with the IPA analysis we also explored the network and pathway analysis of HMGCS2 and other differentially expressed genes in T1D associated cardiac dysfunction for the first time. In agreement with previous studies^25–27^, we found that PDK4, NPPA, ANKRD and SERPINE1 were upregulated in our model and may contribute to cardiac dysfunction in T1D.

We also analyzed down-regulated genes in T1D-associated cardiac dysfunction. Interestingly, Solute Carrier family 41 member 3 (SLC41 A3) was the most significantly down-regulated gene in our study. SLC41 A3 is a recently discovered transmembrane protein that regulates dietary Mg^2+^ intake^28^. However, the role of SLC41A3 in T1D associated with cardiac dysfunction are unknown and needs to be identified.

We performed pathway analysis of all of differentially-expressed genes using the IPA software. The top pathways involved with these genes included ketogensis, ketolysis, glucocorticoid receptor signaling and mevalonate pathway. Ketogenesis is the common phenomenon in T1D diabetic patients who are not able to make insulin^7^. T1D disrupts the balance between ketogenesis and ketolysis, which results in a high ketone level called hyperketonemia^29^. Previous studies also found that these ketone bodies induce oxidative stress which may affect the biological and cellular functions^7, 27, 30^. However, it is still unclear how ketones behave in a cardiac dysfunction with hyperglycemic state. Our data suggest that the T1D triggers expression of many genes that are potentially involved in the ketone body synthesis pathway in the diabetic heart. Therefore, there is a need to reassess the role of ketone bodies synthesis in T1D associated with cardiac dysfunction. There is also need to re-evaluate each molecule that is differentially expressed in our current study to learn how they are involved in ketogenesis pathway.

Based on the 10 differentially expressed genes, we performed a network analysis and defined a network related to ‘amino acid metabolism, small molecule biochemistry and cellular development’ within which six of the 10 characterized genes were placed. This network centers on *SERPINE1*, a serine protease inhibitor that acts as bait for tissue plasminogen activator and that has also been shown to be associated with diabetes^31^. This network also revealed several other interesting regulatory proteins, especially the aryl hydrocarbon receptor nuclear translocator-like protein (*ARNTL*), which serve as a transcription regulator and is involved in circadian function^32^. *ARNTL* has also been associated with hypertension, diabetes, and obesity^32^. Activation of the *ARNTL* could prevent diabetes by increasing Foxp3^+^ regulatory T cells. Our network analysis showed that ARNTL is regulated by different kinase pathways and directly linked with HMGCS2. Previously, Oishi *et al*.^33^ found that circadian clock gene expression is disrupted in peripheral tissues of hypothermic mice fed a ketogenic diet^33^. Therefore, it could be possible that ARNTL may act as transcriptional regulator for HMGCS2 and involved in ketogenesis pathways during the development of T1D associated CVD. However, this statement calls for further study.

Our study also reported the differential expression of several signaling molecule. Many recent studies have shown that ketone bodies induce oxidative stress and upregulate several signaling pathways including ERK1/2, p38MAP, NFkB and Akt^7, 34^. These signaling molecules enhance adhesion molecule expression which results in leukocyte infiltration that leads to tissue damage^7^.

HMGCS2 is generally expressed in liver where it converts acetyl-CoA to ketone bodies^35^. Although the expression of HMGCS2 was detected in extra-hepatic organs, its role is undefined^36, 37^. We found overexpression of HMGCS2 in T1D mice heart tissue with cardiac dysfunction. To explore whether HMGCS2 expression is organ specific or other vital organs also differentially express HMGCS2, we checked the expression of HMGCS2 in heart, liver, kidney and spleen. Our data showed that the expression of HMGCS2 was differentially expressed at both the mRNA and protein level in T1D mice heart tissue. This data confirms our microarray results. Kidney﻿ and heart maintain a metabolic response that controls ketone body utilization^36, 37^. Different experimental approaches have established that cardiac dysfunction is related to changes in cardiac energy metabolism^38–40^. Therefore, it could be possible that HMGCS2 plays a role in cardiac energy metabolism during the cardiac dysfunction related to T1D. However, this hypothesis warrants future study. Although the expression of HMGCS2 was higher in liver but the expression was not different among control and T1D mice. In concordance with our findings, a previous study also did not identify any significant difference of HMGCS2 with diabetic states^24^. We also found that, apart from heart, the expression of HMGCS2 was also significantly elevated in the kidney and spleen. A study found that HMGCS2 expression was abundant in renal glomeruli of diabetic mice. This result further suggested that upregulation of HMGCS2 increased renal ketogenesis which ultimately leads to the diabetic nephropathy that occurs in type 2 diabetes^36^. A literature search did not identify any report where HMGCS2 expression was studied in spleen. We therefore are the first to report that T1D leads to an increase of HMGCS2 expression in spleen tissue.

In conclusion, the present study determined the global expression profile of genes in T1D associated with cardiac dysfunction and conferred their physiological significance. T1D-associated CVD induced a cohort of mRNAs that is associated mainly with ketogenesis and ketolysis pathways. HMGCS2, a ketogenic enzyme was the most up-regulated gene in T1D heart tissue. This result was unanticipated because until now, no such studies have ever reported ketone body synthesis in the diabetic heart. Further studies are required to investigate the molecular mechanisms involved in the up-regulation of HMGCS2 expression in diabetic heart tissue. The ketone body also needs to be assessed during the development of T1D-related cardiac dysfunction.

## Electronic supplementary material

Supplementary Figures

Dataset1

## References

[1] Sarah D. de Ferranti *et al*. Type 1 Diabetes Mellitus and Cardiovascular Disease: A Scientific Statement From the American Heart Association and American Diabetes Association. *Diabetes Care* **37**(10), 2843–2863 (2014). 10.2337/dc14-1720 PMC4170130 25114297

[2] D. M. Maahs *et al*. Cardiovascular Disease Risk Factors in Youth With Diabetes Mellitus: A Scientific Statement From the American Heart Association. *Circulation* **130**(17), 1532–1558 (2014). 10.1161/CIR.0000000000000094 25170098

[3] Jessica L. Harding *et al*. Mortality Trends Among People With Type 1 and Type 2 Diabetes in Australia: 1997–2010. *Diabetes Care* **37**(9), 2579–2586 (2014). 10.2337/dc14-0096 24947787

[4] Eugene Braunwald. Biomarkers in Heart Failure. *New England Journal of Medicine* **358**(20), 2148–2159 (2008). 10.1056/NEJMra0800239 18480207

[5] N. S. Al-Zaid, H. M. Dashti, T. C. Mathew & J. S. Juggi. Low carbohydrate ketogenic diet enhances cardiac tolerance to global ischaemia. *Acta Cardiologica* **62**(4), 381–389 (2007). 10.2143/AC.62.4.2022282 17824299

[6] D. G. Cotter, R. C. Schugar & P. A. Crawford. Ketone body metabolism and cardiovascular disease. *AJP: Heart and Circulatory Physiology* **304**(8), H1060–H1076 (2013). 10.1152/ajpheart.00646.2012 PMC3625904 23396451

[7] Preeti Kanikarla-Marie & Sushil K. Jain. Hyperketonemia and ketosis increase the risk of complications in type 1 diabetes. *Free Radical Biology and Medicine* **95**, 268–277 (2016). 10.1016/j.freeradbiomed.2016.03.020 PMC4867238 27036365

[8] Mukul K. Midha *et al*. Extracting Time-dependent Obese-diabetic Specific Networks in Hepatic Proteome Analysis. *Journal of Proteome Research* 121108101507000 (2012). 10.1021/pr300711a 23050596

[9] H. Ashrafian, M. P. Frenneaux & L. H. Opie. Metabolic Mechanisms in Heart Failure. *Circulation* **116**(4), 434–448 (2007). 10.1161/CIRCULATIONAHA.107.702795 17646594

[10] L. Cai *et al*. Inhibition of Superoxide Generation and Associated Nitrosative Damage Is Involved in Metallothionein Prevention of Diabetic Cardiomyopathy. *Diabetes* **54**(6), 1829–1837 (2005). 10.2337/diabetes.54.6.1829 15919806

[11] Marie Paschaki *et al*. Transcriptomic Analysis of Murine Embryos Lacking Endogenous Retinoic Acid Signaling. *PLoS ONE*8(4), e62274 (2013). 10.1371/journal.pone.0062274 PMC3634737 23638021

[12] C. Thangavel *et al*. RB Loss Promotes Prostate Cancer Metastasis. *Cancer Research* **77**(4), 982–995 (2017). 10.1158/0008-5472.CAN-16-1589 PMC5700768 27923835

[13] Ebun Omoyinmi *et al*. Mitochondrial and oxidative stress genes are differentially expressed in neutrophils of sJIA patients treated with tocilizumab: a pilot microarray study. *Pediatric Rheumatology* **14**(1) (2016). 10.1186/s12969-016-0067-7 PMC4746827 26861863

[14] A. Vila-Brau, A. L. De Sousa-Coelho, C. Mayordomo, D. Haro & P. F. Marrero. Human HMGCS2 Regulates Mitochondrial Fatty Acid Oxidation and FGF21 Expression in HepG2 Cell Line. *Journal of Biological Chemistry* **286**(23), 20423–20430 (2011). 10.1074/jbc.M111.235044 PMC3121469 21502324

[15] E. T. Cullingford *et al*. Molecular cloning of rat mitochondrial 3-hydroxy-3-methylglutaryl-CoA lyase and detection of the corresponding mRNA and of those encoding the remaining enzymes comprising the ketogenic 3-hydroxy-3-methylglutaryl-CoA cycle in central nervous system of suckling rat. *Biochemical Journal* **329**(2), 373–381 (1998). 10.1042/bj3290373 PMC1219054 9425122

[16] F. G. Hegardt. Mitochondrial 3-hydroxy-3-methylglutaryl-CoA synthase: a control enzyme in ketogenesis. *Biochemical Journal* **338**(3), 569–582 (1999). PMC1220089 10051425

[17] Katz M, Giani E, Laffel L (2015). Challenges and Opportunities in the Management of Cardiovascular Risk Factors in Youth With Type 1 Diabetes: Lifestyle and Beyond. Current Diabetes Reports.

[18] Styskal J, Van Remmen H, Richardson A, Salmon A (2012). Oxidative stress and diabetes: What can we learn about insulin resistance from antioxidant mutant mouse models?. Free Radical Biology and Medicine.

[19] Sugamura K, Keaney JF (2011). Reactive oxygen species in cardiovascular disease. Free Radical Biology and Medicine.

[20] Huynh K, Bernardo BC, McMullen JR, Ritchie RH (2014). Diabetic cardiomyopathy: Mechanisms and new treatment strategies targeting antioxidant signaling pathways. Pharmacology & Therapeutics.

[21] Nakamura MT, Yudell BE, Loor JJ (2014). Regulation of energy metabolism by long-chain fatty acids. Progress in Lipid Research.

[22] Mascaro C, Buesa C, Ortiz JA, Hare D, Hegardt FG (1995). Molecular Cloning and Tissue Expression of Human Mitochondrial 3-Hydroxy-3-Methylglutaryl-CoA Synthase. Archives of Biochemistry and Biophysics.

[23] Suzuki J (2001). Absence of cardiac lipid accumulation in transgenic mice with heart-specific hsl overexpression. American journal of physiology. Endocrinology and metabolism.

[24] Cook GA, Lavrentyev EN, Pham K, Park EA (2017). Streptozotocin diabetes increases mRNA expression of ketogenic enzymes in the rat heart. Biochimica et Biophysica Acta (BBA) – General Subjects.

[25] Chambers KT (2011). Chronic Inhibition of Pyruvate Dehydrogenase in Heart Triggers an Adaptive Metabolic Response. Journal of Biological Chemistry.

[26] Hunt KJ (2015). Longitudinal Association Between Endothelial Dysfunction, Inflammation, and Clotting Biomarkers With Subclinical Atherosclerosis in Type 1 Diabetes: An Evaluation of the DCCT/EDIC Cohort. Diabetes Care.

[27] Jain SK, McVie R (1999). Hyperketonemia can increase lipid peroxidation and lower glutathione levels in human erythrocytes in vitro and in type 1 diabetic patients. Diabetes.

[28] de Baaij JHF (2013). Elucidation of the distal convoluted tubule transcriptome identifies new candidate genes involved in renal Mg2+ handling. AJP: Renal Physiology.

[29] Sassa M (2008). Glycemic instability in type 1 diabetic patients: Possible role of ketosis or ketoacidosis at onset of diabetes. Diabetes Research and Clinical Practice.

[30] Pelletier A, Coderre L (2007). Ketone bodies alter dinitrophenol-induced glucose uptake through AMPK inhibition and oxidative stress generation in adult cardiomyocytes. AJP: Endocrinology and Metabolism.

[31] Kaur P, Reis MD, Couchman GR, Forjuoh SN, Greene JF (2010). SERPINE 1 Links Obesity and Diabetes: A Pilot Study. Journal of Proteomics & Bioinformatics.

[32] Eckel-Mahan K, Sassone-Corsi P (2013). Metabolism and the Circadian Clock Converge. Physiological Reviews.

[33] Oishi K, Yamamoto S, Uchida D, Doi R (2013). Ketogenic diet and fasting induce the expression of cold-inducible RNA-binding protein with time-dependent hypothermia in the mouse liver. FEBS Open Bio.

[34] Abdelmegeed MA (2004). Acetoacetate Activation of Extracellular Signal-Regulated Kinase 1/2 and p38 Mitogen-Activated Protein Kinase in Primary Cultured Rat Hepatocytes: Role of Oxidative Stress. Journal of Pharmacology and Experimental Therapeutics.

[35] Li J, Viswanadha S, Loor JJ (2012). Hepatic Metabolic, Inflammatory, and Stress-Related Gene Expression in Growing Mice Consuming a Low Dose of -10, -12-Conjugated Linoleic Acid. Journal of Lipids.

[36] Zhang D (2011). Proteomics analysis reveals diabetic kidney as a ketogenic organ in type 2 diabetes. AJP: Endocrinology and Metabolism.

[37] Wentz AE (2010). Adaptation of Myocardial Substrate Metabolism to a Ketogenic Nutrient Environment. Journal of Biological Chemistry.

[38] Ingwall JS (2004). Is the Failing Heart Energy Starved?: On Using Chemical Energy to Support Cardiac Function. Circulation Research.

[39] Lopaschuk GD (2006). Optimizing Cardiac Fatty Acid and Glucose Metabolism as an Approach to Treating Heart Failure. Seminars in Cardiothoracic and Vascular Anesthesia.

[40] Stanley WC (2005). Myocardial Substrate Metabolism in the Normal and Failing Heart. Physiological Reviews.

