# Supplementary material for: HMGCS2 is a key ketogenic enzyme potentially involved in type 1 diabetes with high cardiovascular risk
Source: Sci Rep. 2017 Jul 4;7:4590. doi: 10.1038/s41598-017-04469-z (PMC5496911; doi:10.1038/s41598-017-04469-z)

Supplementary figures

HMGCS2 is a key ketogenic enzyme potentially involved in Type 1 diabetes with high cardiovascular risk.

Sanket Kumar Shukla[1a], Weijing Liu[1b], Kunal Sikder[a], Sankar Addya[c], Amrita Sarkar[a], Yidong Wei[d], Khadija Rafiq[a]*

Department of Medicine, Center of Translational Medicine, Thomas Jefferson University, Philadelphia, PA-19107, USA.[a]

Internal Medicine-Cardiovascular Department, Shanghai Tenth People's Hospital Middle of Yanchang Road, Zhabei district, Shanghai, China.[b]

Kimmel Cancer Centre, Thomas Jefferson University, Philadelphia, PA-19107, USA.[c]

Department of Cardiology, Shanghai Tenth People's Hospital of Tongji University 301 Yanchang Road, Shanghai, China.[d]

*** Corresponding author**

Khadija Rafiq, PhD
Assistant Professor
Center for Translational Medicine, Department of Medicine
Thomas Jefferson University, Rm 543A Jefferson Alumni Hall
1020 Locust Street
Philadelphia, PA 19107
215-503-5593
Khadija.Rafiq@jefferson.edu

Figure S1: The 2nd most top network, with an IPA score of 7. Cellular Development, Hematological System Development and Function, Hematopoiesis

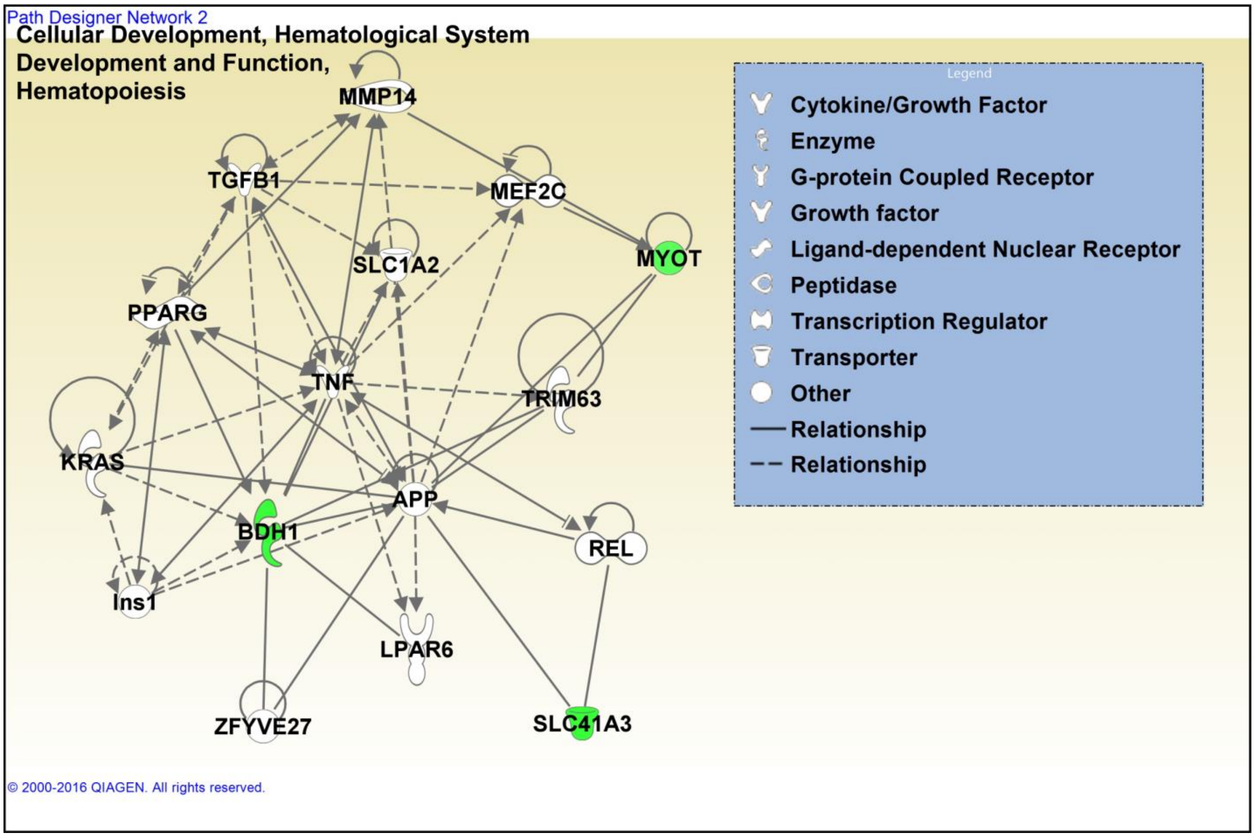

Figure S2: β-hydroxy butyrate level (ketone bodies) in serum sample of mice model.

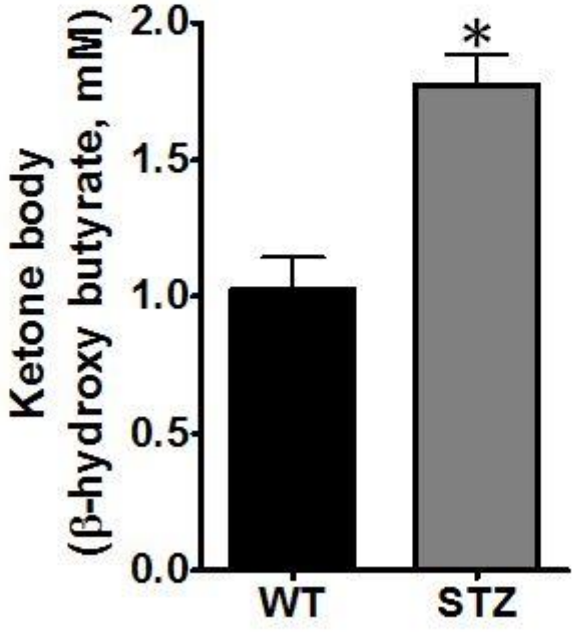

Supplement: Supplementary file 1 — Supplementary Figures [file 41598_2017_4469_MOESM1_ESM.pdf]
